# Dietary Cadmium Chloride Supplementation Impairs Renal Function and Bone Metabolism of Laying Hens

**DOI:** 10.3390/ani9110998

**Published:** 2019-11-19

**Authors:** Mingkun Zhu, Wenting Zhou, Luhong Bai, Huaiyu Li, Liansong Wang, Xiaoting Zou

**Affiliations:** 1 Key Laboratory of Animal Feed and Nutrition of Zhejiang Province, Key Laboratory of Animal Nutrition and Feed Science in East China, Ministry of Agriculture, The Key Laboratory of Molecular Animal Nutrition, Ministry of Education, College of Animal Sciences, Zhejiang University, Hangzhou 310058, China; mkzhu@zju.edu.cn (M.Z.); 21817022@zju.edu.cn (W.Z.); 21817070@zju.edu.cn (L.B.); 21817082@zju.edu.cn (H.L.); 2 Jiande Weifeng Feed Co., Ltd., Hangzhou 311603, China; myronzju@163.com

**Keywords:** cadmium, renal function, bone metabolism, histopathology, laying hens

## Abstract

**Simple Summary:**

In this study, the effects of cadmium (Cd) on renal function and bone metabolism of laying hens were investigated. Results indicated that supplementation with Cd can damage renal function, induce oxidative stress in the kidney, disrupt the body’s calcium balance and hormone secretion, and negatively affect bone metabolism of laying hens. The results demonstrated that chronic exposure to Cd could induce renal dysfunction and bone damage in laying hens.

**Abstract:**

This study was conducted to evaluate the toxic effects of cadmium (Cd) on the kidney function and bone development in laying hens. A total of 480 Hy-line laying hens aged 38 weeks were randomly allocated into five treatments, each of which included six replicates of 16 birds. The concentrations of Cd in the diets of the five groups were 0.47, 7.58, 15.56, 30.55, and 60.67 mg/kg. Results showed that serum calcium (Ca) levels decreased significantly in the 60.67 mg Cd/kg diet group (*p* < 0.05). The activities of serum alkaline phosphatase (ALP) and bone ALP (BALP) decreased significantly in the 15.56, 30.55 and 60.67 mg Cd/kg diet groups (*p* < 0.05). The levels of parathyroid hormone (PTH) increased significantly in the 30.55 and 60.67 mg Cd/kg diet groups, and the estradiol (E2), 1,25-(OH)2-D3 and calcitonin (CT) decreased significantly with the increase of dietary Cd supplementation (*p* < 0.05). Histological results presented enlargements of renal tubules and tubular fibrosis in the kidney and decreased trabecular bone in the tibia. Tartrate-resistant acidic phosphatase (TRAP) staining results of tibia showed that osteoclast was significantly increased at the relatively high dose of dietary Cd (*p* < 0.05). In addition, the renal function indicators of blood urea nitrogen (BUN), urea acid (UA), and creatinine were significantly increased in Cd supplemented groups compared with the control group (*p* < 0.05). Low dose Cd exposure induced antioxidant defenses accompanying the increase in activities of catalase (CAT), glutathione peroxidase (GSH-Px), and the levels of glutathione (GSH) in renal tissue. At the same time, with the increased Cd levels, the activities of CAT, GSH-Px decreased significantly, and the level of malondialdehyde (MDA) increased significantly (*p* < 0.05). The activities of Na+/K+-ATPase and Ca2+/Mg2+-ATPase decreased significantly in the relatively high levels of dietary Cd (*p* < 0.05). These results suggest that Cd can damage renal function and induce disorders in bone metabolism of laying hens.

## 1. Introduction

Cadmium (Cd) is a ubiquitous and a non-biodegradable pollutant with a very long biological half-life and is ranked the seventh toxicant in the priority list of hazardous substances of the Agency for Toxic Substances and Disease Registry (ATSDR) [1]. Chronic Cd toxicity is considered a significant health concern. Long-term exposure to Cd can result in a variety of adverse effects on biomolecules and different organs. Among them, kidneys and bones are the two major target organs of Cd-induced toxicity [2,3]. In the general population, Cd exposure mainly results in an injury of the proximal tubular epithelial cells, where Cd is selectively accumulated [4]. The effects of Cd on bone are generally observed at a later period and occur primarily due to the consequences of Cd nephrotoxicity [5]. Accumulation of Cd can lead to renal failure, and perturbation of the vitamin D metabolic pathway, which are associated with several health problems including osteoporosis or osteomalacia [6]. However, many studies also found that the toxic effect of Cd on bone metabolism might occur concomitantly with nephrotoxicity [3]. Under certain conditions, Cd exposure at levels that do not impair renal function can directly affect bone development, leading to early bone loss [7]. However, Horiguchi et al. [8] reported that environmental exposure to cadmium at a level insufficient to induce renal tubular dysfunction does not affect bone density among female Japanese farmers. Therefore, it is likely that Cd-induced bone injury is a secondary effect of Cd nephrotoxicity [5,8].

In birds, bone not only provides structural support but is also an important mineral source and this latter role is possibly more important than the former one [9]. Many studies have demonstrated that long-term and low-level exposure to Cd could reduce the absorption and reabsorption of calcium (Ca) from intestines and renal tubules, and increase urinary Ca excretion [10]. Ca contributes over a range of different functions in animals and humans, including being essential for bone mineralization and eggshell deposition in birds [11]. In addition, bone formation is regulated by changes in circulating hormone levels. Squire et al. [12] indicated that estrogen (E2) treatment affected the quantity of medullary bone, which contributes to eggshell formation, in female house finches. Parathyroid hormone (PTH) and calcitonin (CT) are also involved in 1,25-dihydroxyvitamin D3 (1,25(OH)_2_D_3_) production in the kidney, and ultimately affect bone metabolism [13,14].

Therefore, there may be connections between a Cd contaminated diet and general histopathological damages to bone and kidney of laying hens that warrant further investigation. By using diets contaminated with varying levels of Cd, we investigated the toxic effects of Cd on hormone levels, bone metabolism, and renal function in laying hens.

## 2. Materials and Methods

### 2.1. Animal Ethics

The experiment was carried out according to the Guiding Principles in the Use of Animals in Toxicology, adopted by the Chinese Society of Toxicology. The experimental procedures were approved by the Animal Ethics Committee of the Zhejiang University (No. ZJU2013105002), Hangzhou, China.

### 2.2. Birds, Diets, and Management

A total of 480 Hy-line brown laying hens, with an age of 38 weeks, was obtained from a commercial poultry layer farm of Jiande in China. CdCl_2_ was purchased from Sinopharm Chemical Reagent Co., Ltd. (Shanghai, China; purity ≥99%). After three days of acclimation, hens were randomly assigned into 5 treatments, each of which included 6 replicates of 16 laying hens. The protocol of treatments was as follows: 1. Basal diet (control); 2. Basal diet + 7.5 mg/kg Cadmium (Cd); 3. Basal diet + 15 mg/kg Cd; 4. Basal diet + 30 mg/kg Cd; 5. Basal diet + 60 mg/kg Cd. During the whole experimental period (10 weeks, including a 1-week adaptation period and a 9-week experimental stage), diets and water were provided ad libitum, birds were kept in a naturally ventilated poultry house with temperature ranging from 23 to 26 °C, the relative humidity was between 65% and 75%, and the light regime was 16 h light/day (20 lx). All the animals were appropriately treated according to the criteria outlined in the “Guide for the Care and Use of Laboratory Animals” prepared by the National Academy of Science and published by the National Institute of Health. The compositions and analysis of basal diets are presented in Table 1. The actual concentrations of dietary Cd were detected by graphite furnace atomic absorption spectrometry (GFAAS) (Perkin-Elmer Analyst 200, Boston, MA, USA) and were 0.47, 7.58, 15.56, 30.55, and 60.67 mg/kg, respectively.

### 2.3. Collection of Samples and Measurement

At the end of the experimental period, nine weeks after the beginning of Cd dietary supplementation, 12 birds per treatment (2 birds each replicate) were randomly selected. After the hens were fasted for 12 h (water was offered ad libitum), blood samples were collected in 1.5 mL Eppendorf tubes by puncture of the wing vein. These tubes were put on ice to allow the blood to clot and were immediately centrifuged at 3000× *g* for 15 min to separate serum and stored at −80 °C for biochemical and hormonal analysis. Serum contents of Ca, phosphorus (P), renal function indicators including uric acid (UA), creatinine and blood urea nitrogen (BUN), along with the activity of alkaline phosphatase (ALP) were determined in serum samples using commercially available assay kits (Cat No. C004-2-1, C006-1-1, C012-1-1, C011-1-1, C013-2-1, and A059-1-1, respectively; Nanjing Jiancheng Bioengineering Institute, Nanjing, China) according to the instructions of the manufacturer. The concentration of hormones including 1,25-(OH)2-D3, PTH, CT, estradiol (E2) and bone alkaline phosphatase (BALP) were determined by using appropriate ELISA kits (Enzyme-linked Biotechnology ELISA kits, Shanghai, China; Cat No. ml714212, ml071028 and ml728931 for 1,25- (OH)_2_-D_3_, PTH, and CT, respectively; Nanjing Jiancheng Bioengineering Institute ELISA kits, Nanjing, China; Cat No. H234 and H102 for BALP and E2, respectively).

Birds were euthanized by cervical dislocation. Samples of kidney were dissected and kept at −20 °C for the determination of Cd residues. The residues of Cd in serum and kidney were detected by the GFAAS (Perkin-Elmer Analyst 200, Boston, MA, USA). Three portions of kidney were snap frozen in liquid nitrogen and stored at −80 °C for antioxidant analysis. Kidney tissue samples were cut into small pieces (about 0.1 g), and then were mixed to ice cold physiological saline at a ratio of 1:9 to prepare a 10% tissue homogenate. The homogenate was centrifuged at 3000 rpm for 10 min at 4 °C. The supernatant was collected and stored at −80 °C until further analysis. The activities of catalase (CAT), glutathione peroxide (GSH-Px), ATPase (Na^+^-K^+^-ATPase and Ca^2+^-Mg^2+^-ATPase), and the contents of glutathione (GSH) and malondialdehyde (MDA) in the renal supernatants were assessed using commercially available assay kits (Cat No. A007-1-1, A005-1-1, A016-2-2, A005-1-2, and A003-1-1, respectively; Nanjing Jiancheng Bioengineering Institute, Nanjing, China) according to the instructions of the manufacturer.

Portion of kidney and tibia were selected and inserted in 4% paraformaldehyde for the histopathological and immunohistochemistry analysis. The kidney tissues were fixed with 4% paraformaldehyde then trimmed and embedded in paraffin wax. The paraffin sections were cut 5–6 µm thick using a microtome (RM2016, Leica Microsystems GmbH, Wetzlar, Germany), then stained with hematoxylin and eosin (H & E) for histopathological observation by optical microscopy at a final magnification of 400× (Nikon Eclipse 80i, Nikon, Tokyo, Japan). In addition, the tibias of laying hens were fixed in 4% paraformaldehyde, followed by decalcification in ethylenediaminetetra-acetic acid (EDTA). Then, tibias were dehydrated through a series of ascending ethanol solutions (70% to 100%), cleared with dimethylbenzene, embedded in paraffin wax, and sliced at about 8 µm with a microtome for H & E staining. For tartrate-resistant acid phosphatase (TRAP) staining, serial 4 µm thick tibia sections were prepared and stained to identify osteoclasts as described previously [15]. The same birds were used for blood, kidney, and tibia sampling.

### 2.4. Statistical Analysis

Data were statistically analyzed by one-way ANOVA using SPSS 20.0 for Windows (SPSS Inc., Chicago, IL, USA). Significant differences among treatment means were determined using Tukey post-hoc tests. Differences were considered statistically significant when the possibility value (*p*-value) was less than 0.05 (*p* < 0.05) unless noted otherwise.

## 3. Results

### 3.1. Cd Residues and Ca, P Levels in the Serum and Kidney of Laying Hens

**Figure 1:**
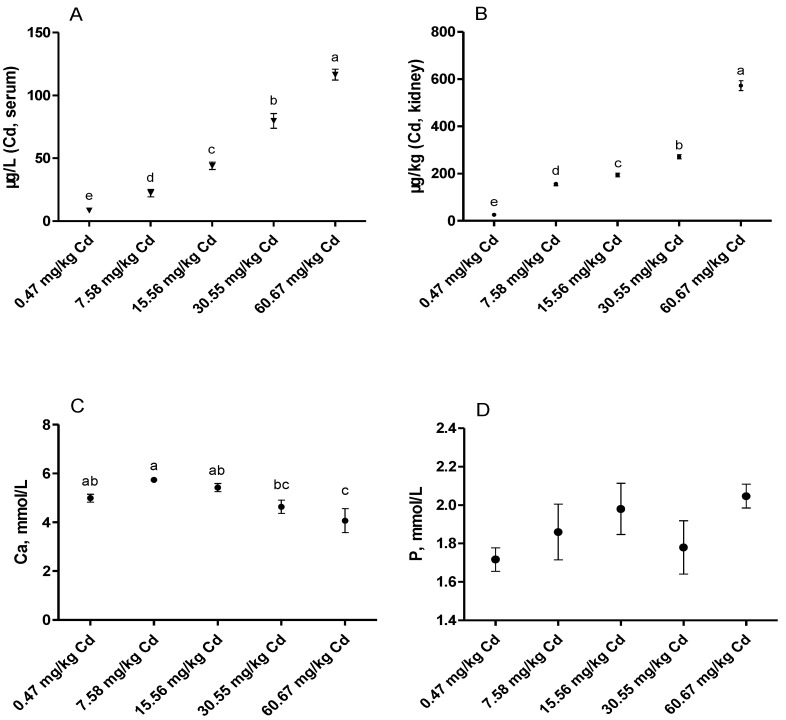
Effects of Cd on Cd, Ca and P levels in the serum and kidney of laying hens. (**A**) serum Cd level; (**B**) Cd level in the kidney; (**C**) serum Ca level; (**D**) serum P level. Values are presented as the mean ± SE (*n* = 8). ^a–e^ Means with different superscript letters are significantly different (*p* < 0.05).

The Cd residues and Ca and P levels in the serum of laying hens are presented in Figure 1. With an increase of dietary Cd concentration, the levels of Cd both in the serum and kidney increased linearly (*p* < 0.05). When compared with the control group, the serum Ca level was significantly decreased in the group of 60.67 mg/kg Cd (*p* < 0.05). However, there was no significant difference in the serum P level among the five groups (*p* > 0.05).

### 3.2. ALP Activity in the Serum of Laying Hens

**Figure 2:**
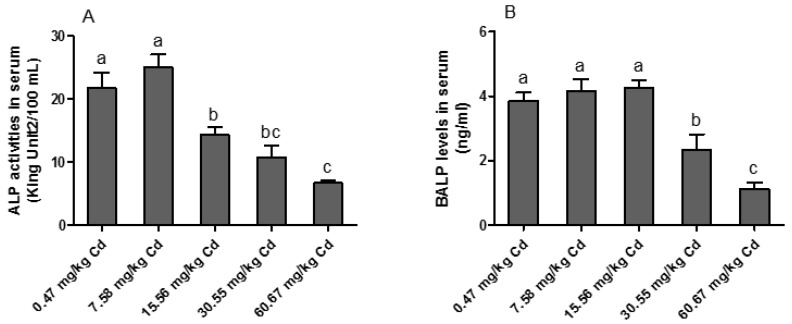
Effects of Cd on the activity of alkaline phosphatase (ALP) and the level of bone ALP (BALP) in the serum of laying hens. (**A**) ALP activity in the serum; (**B**) BALP level in the serum. Values are presented as the mean ± SE (*n* = 8). ^a–c^ Means with different superscript letters are significantly different (*p* < 0.05).

As illustrated in Figure 2, the activity of ALP in serum was significantly decreased in the groups of 15.56, 30.55 and 60.67 mg/kg Cd (*p* < 0.05) as compared with the other groups. Similarly, the concentration of BALP in the serum was significantly decreased in the groups of 30.55 and 60.67 mg/kg Cd (*p* < 0.05) as compared with the other groups.

### 3.3. Hormone Levels in the Serum of Laying Hens

**Figure 3:**
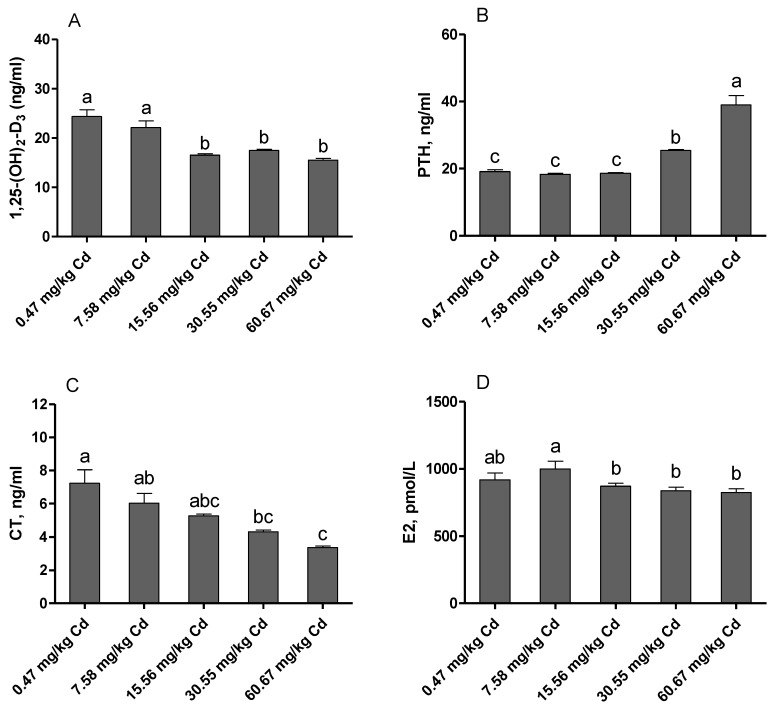
Effects of Cd on hormone levels in serum of laying hens. (**A**) 1,25-(OH)_2_-D_3_, 1,25- dihydroxyvitamin D3; (**B**) PTH, Parathyroid Hormone; (**C**) CT, calcitonin; (**D**) E2, Estradiol. Values are presented as the mean ± SE (*n* = 8). ^a–c^ Means with different superscript letters are significantly different (*p* < 0.05).

The hormone levels in the serum of laying hens exposed to Cd are displayed in Figure 3. The levels of E2, calcitonin (CT), and 1,25-(OH)2-D3 were significantly decreased in the relatively high Cd levels supplemented groups (15.56 or/and 30.55 and 60.67 mg/kg Cd) as compared with the control group (*p* < 0.05). In contrast, the levels of PTH were significantly increased in the groups of 30.55 and 60.67 mg/kg Cd as compared with other groups (*p* < 0.05).

### 3.4. Bone (Tibia) Volume of Laying Hens Exposed to Cd

**Figure 4:**
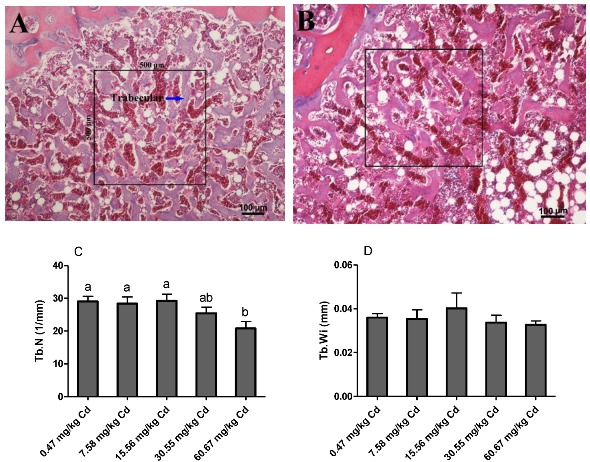
Photomicrographs of hematoxylin and eosin (H & E) stained (100× magnification, bar = 100 μm, *n* = 6) longitudinal sections of the tibia showing the decrease in bone volume observed in Cd (60.67 mg/kg group) exposed animals (**B**) as compared to controls (0.47 mg/kg Cd group) (**A**). (**C**) trabecular number; (**D**) trabecular width. Values are presented as the mean ± SE (*n* = 6). ^a–b^ Means with different superscript letters are significantly different (*p* < 0.05).

The results of the histopathological changes in the tibia of hens are shown in Figure 4. No histopathological alterations were observed in the tibia of laying hens in the control and low dose Cd supplemented groups (*p* > 0.05). However, with the increase of dietary Cd levels the trabecular space was widened, and the trabecular number (Tb. N, 1/mm) was decreased, and this was statistically significant in the group of 60.67 mg/kg Cd (*p* < 0.05). There were no significant changes in the trabecular width (Tb. Wi) among the five groups (*p* > 0.05).

### 3.5. Histological Evaluation of Osteoclast

**Figure 5:**
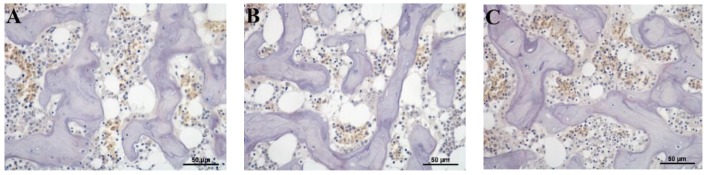

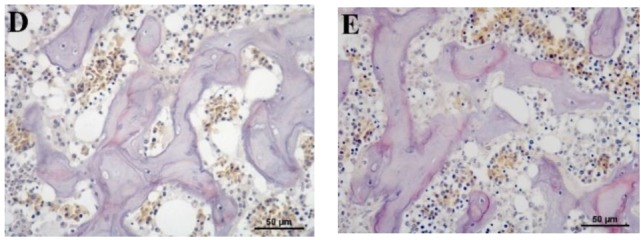
Effects of Cd on the osteoclast activity of tibias in laying hens. Tartrate-resistant acid phosphatase-stained (TRAP-stain) tibial bone sections (400× magnification, bar = 50 μm, *n* = 6). (**A**) Control (Cadmium 0.47 mg/kg); (**B**) Cadmium 7.58 mg/kg; (**C**) Cadmium 15.56 mg/kg; (**D**) Cadmium 30.55 mg/kg; (**E**) Cadmium 60.67 mg/kg.

In order to evaluate the toxic effects of Cd on osteoclastogenesis, a TRAP-stained assay was designed to determine osteoclast activity and the results are shown in Figure 5. When compared with the control group, the relatively low dose Cd addition (7.58 and 15.56 mg/kg Cd) did not have adverse effects on osteoclastogenesis in the tibia. However, with the further increased supplementation of dietary Cd (30.55 and 60.67 mg/kg Cd), the TRAP staining assay showed that osteoclasts increased significantly as compared with other groups.

### 3.6. Renal Function of Laying Hens

At the same time, the effects of a Cd contaminated diet on the renal function of laying hens were examined. The results presented in Table 2 indicated that with increased dietary Cd levels, the contents of UA, creatinine, and urea nitrogen (UN) in the serum of laying hens all trended to increase, and this was statistically significant in the group of 60.67 mg/kg Cd (*p* < 0.05).

### 3.7. Histopathological Variations of the Kidney

**Figure 6:**
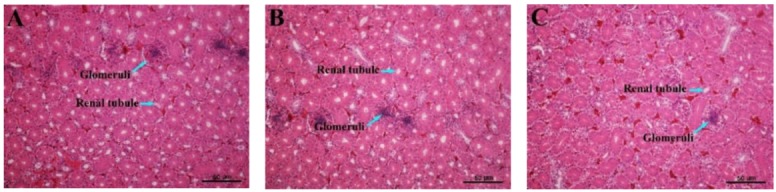

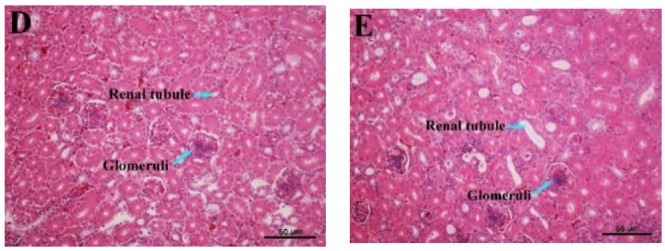
Renal histomorphology from various experimental groups of laying hens. The kidney sections were stained with hematoxylin and eosin (H & E, 100× magnification). (**A**) Control (Cadmium 0.47 mg/kg); (**B**) Cadmium 7.58 mg/kg; (**C**) Cadmium 15.56 mg/kg; (**D**) Cadmium 30.55 mg/kg; (**E**) Cadmium 60.67 mg/kg.

The results of the histopathological changes in the kidney are shown in Figure 6 and reveal significant damage to the kidney tissue of laying hens with increased dietary Cd supplementation. The photomicrographs of the control group (0.47 mg/kg Cd) and low dosage of Cd supplemented group (7.58 mg/kg Cd) show evidence of apparently normal kidney histoarchitecture. However, with the increase of dietary Cd levels, kidney tissues presented a shrinkage of the glomeruli, enlargements of the renal tubules, and tubular fibrosis. In addition, the glomeruli were characterized by the disorganization of the mesangial matrix in the Cd supplemented groups of 15.56, 30.55, and 60.67 mg/kg.

### 3.8. Renal Antioxidant Parameters and ATPase Activities

**Figure 7:**
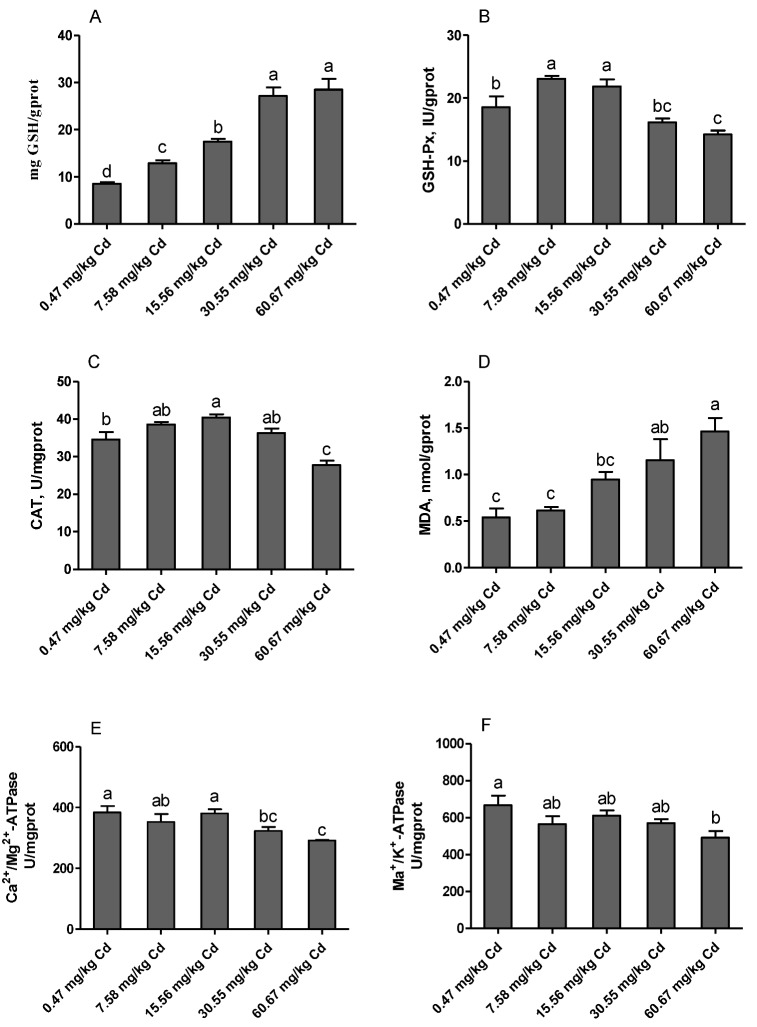
Effects of Cd on the activities of antioxidant enzymes and ATPase in kidney of laying hens. (**A**) GSH, glutathione; (**B**) GSH-Px, glutathione peroxidase; (**C**) CAT, catalase; (**D**) MDA, malondialdehyde; (**E**) Ca^2^^+^/Mg^2+^-ATPase; (**F**) Na^+^/K^+^-ATPase. Values are presented as the mean ± SE (*n* = 8). ^a–d^ Means with different superscript letters are significantly different (*p* < 0.05).

The results from the current experiment revealed that feeding laying hens with a diet contaminated with Cd has negative effects on the antioxidant status and cell homeostasis of the kidney (Figure 7). The levels of GSH increased significantly with the increased dietary Cd (*p* < 0.05). The activities of GSH-Px and CAT increased significantly in the groups of 7.58 and/or 15.56 mg/kg Cd, and then decreased with the increased dietary Cd addition, and this was statistically significant in the group of 60.67 mg/kg Cd as compared with the control (*p* < 0.05). The content of MDA was increased with the increase of dietary Cd levels, and significantly in the groups of 30.55 and 60.67 mg/kg Cd as compared with control (*p* < 0.05). In addition, the activities of ATPase, including Na^+^/K^+^-ATPase and Ca^2+^/Mg^2+^-ATPase were significantly decreased in the group of 60.67 mg/kg Cd as compared with the control group (*p* < 0.05).

## 4. Discussion

Levels of Ca, inorganic P, and ALP in blood are valuable indicators of mineral status and bone mineralization [16]. Ca and P play an important role in the homeostasis of the body and ensure appropriate conditions for a variety of biological activities such as energy utilization, signal transduction, nucleic acid synthesis, and bone mineralization. In poultry, Ca has a critical biological role in the development and strengthening of bone [17]. Studies reported that chronic exposure to Cd could induce hypocalcemia by inhibiting the Ca^2+^-ATPase of the branchial gill in tilapia fish [18,19]. Liao et al. [20] found that exposure to Cd could disturb the balance of Ca and P in the body of ducks. Similarly, in the current study, we found that with increased dietary Cd, the contents of Cd in the serum increased linearly, and the serum Ca concentrations were significantly decreased in the group of 60.67 mg/kg Cd. Cd and Ca are divalent metals with similar chemical properties [21]. In vivo, Cd may induce hypocalcemia by blocking the intracellular Ca^2+^ efflux, decreasing intestinal absorption of Ca^2+^ and renal Ca^2+^ reabsorption, and increasing excretion of urinary Ca^2+^ [22,23,24]. However, no significant changes in the levels of P were observed. These results suggest that Cd disturbed Ca homeostasis in laying hens, which might be attributed to Cd disturbance of the absorption and reabsorption function of the intestine and renal tubules.

In addition, ALP plays an important role in bone formation and mineralization [25]. The total activity of ALP in serum can be regarded as an indicator of levels of local ALP isoforms in many tissues, including that in bone and liver that constitute about 95% of the total ALP activity in serum, of which each account for about 50% [26]. BALP, as the bone-specific isoform of ALP, is a bone formation marker that is found in the surface of osteoblasts. Our results showed that diets contaminated with Cd significantly decreased the activity of total ALP in serum. One possible explanation is that Cd can substitute for Zn or interact with nucleophilic ligands, essential for enzymatic activity resulting in a blockage of ALP activity, as previously reported by Treviño et al. [27]. Another explanation is that Cd can reduce ALP secretion by inhibiting osteoblast viability and diminishing ossification [28]. At the same time, low calcium intake caused by Cd intoxication appeared to affect bone metabolism.

PTH, CT, and 1,25-(OH)_2_-D_3_, the main calciotropic hormones, are known to regulate the balance of Ca and P, and affect bone metabolism and calcification [29,30,31]. The increased secretion of PTH is in response to low blood Ca^2+^ levels and stimulates bone resorption and renal tubular Ca^2+^ reabsorption [14]. In contrast, CT, released in response to elevated blood Ca^2+^, is a potent inhibitor of bone resorption [32]. In the current study, the content of CT in serum decreased with the increase of dietary Cd supplementation and was significant in the groups of 30.55 and 60.67 mg/kg Cd, while, the concentration of PTH increased significantly in both groups. The enhanced PTH and reduced CT levels in serum in accordance with the lower Ca concentration seem to suggest that administration of relatively high Cd doses results in changes in bone mineral status. In addition, 1,25-(OH)_2_-D_3_, which is produced by the mitochondria of the proximal tubular cells, mainly increases the small intestine Ca and P absorption, and maintains the plasma concentrations of Ca and P [33]. Studies have reported that accumulation of Cd in the renal cortex could interfere with conversion of 25-hydroxy vitamin D (25(OH)D) to the bioactive form 1,25-(OH)_2_-D_3_ [34]. In the present study, we observed that the content of 1,25-(OH)_2_-D_3_ in serum decreased significantly with the increase of dietary Cd addition. It seems possible that the decreased serum content of 1,25-(OH)_2_-D_3_, and disturbance of Ca and P balance are associated with Cd-induced renal dysfunction. Many studies also found that estrogen deficiency in ovariectomized animals resulted in a substantial decrease in bone mass [35]. Parikka et al. [36] reported that estrogen reduced the depth of resorption pits by disturbing the organic bone matrix degradation activity of mature osteoclasts. In addition, birds treated with E2 presented a direct or indirect influence vitamin D3 metabolism [37]. Our results showed that the concentration of E2 in serum decreased significantly in the groups of 30.55 and 60.67 mg/kg Cd. These results indicated that chronic exposure to Cd affected the metabolism and proper function of calciotropic hormones in laying hens.

Observations of tibia showed that exposure to Cd caused a decrease in the number of trabecular but did not affect the trabecular width. Similarly, Rodríguez and Mandalunis reported that Wistar rats exposed to Cd presented a significant decrease in the trabecular number in the tibia and an increase in the activity of TRAP+ osteoclast [38]. In the current study, the result of TRAP staining also showed that osteoclasts increased significantly in the relatively high-dose Cd exposed groups (30.55 and 60.67 mg/kg Cd). In in vitro studies, Wilson et al. [39] found that Cd accelerated the differentiation of new osteoclasts from progenitor cells and increased the mature osteoclast activity. The enhanced activity of osteoclasts and decreased trabecular number confirmed that exposure to Cd could induce bone damage in laying hens, which might be an effect of decreased bone formation and increased bone resorption.

Many studies indicated that, in chronic Cd exposure situations, bone diseases were usually accompanied by kidney damage [5,8]. As discussed above, we pointed out that Cd-induced renal dysfunction might be one of the causes of decreased 1,25-(OH)_2_-D_3_ synthesis and imbalance of Ca and P homeostasis. Our results showed that creatinine, uric acid, and urea nitrogen levels in serum increased with the increase of dietary Cd supplementation, and significantly in the groups of 30.55 and/or 60.67 mg/kg Cd. The serum levels of urea, uric acid and creatinine can be used as an indicator of renal dysfunction and damage [40]. Our results further confirmed that administration of Cd induced shrinkage of glomeruli, enlargements of renal tubules, and tubular fibrosis. Brzóska et al. [41] indicated that Cd could cause damage in the structure and function of kidneys even at a relatively low level (5 mg/L) corresponding to human environmental exposure, and the target for Cd action is the tubules including proximal convoluted tubules and straight tubules. The damage of proximal tubules finally results in transport defects [42,43]. Consequently, it can be concluded that Cd exposure is associated with renal dysfunction, which is an important factor related to bone damage in laying hens.

Induction of oxidative stress is considered an important mechanism of Cd toxicity. The role of Cd induced oxidative stress in various cells and organs of the body through the disruption of oxidant/antioxidant balance and the deterioration of antioxidant enzymes activities is well documented [44,45]. In the kidney, Cd^2+^ is mainly taken up by proximal tubule cells, in which it can induce oxidative stress and ROS formation by displacing redox-active elements, damaging mitochondria, or scavenging GSH [46]. GSH has been found to be involved in attenuating renal toxicity through displacing metallothionein (MTs) from the complex of CdMT [47]. In addition, GSH is also a coenzyme for GSH-Px, which plays an important role both in converting H_2_O_2_ to water and removing lipid hydroperoxides [48]. In the current study, we found that the content of GSH increased significantly with the increase of dietary Cd supplementation. This finding is consistent with the reports made by Nair et al. [49], who stated that both acute exposure to higher Cd^2+^ levels or chronic exposure to low Cd^2+^ concentration increased the GSH content. At the same time, increased activities of GSH-Px and CAT in the relatively low Cd dose groups were observed. A possible explanation is that the low dose of Cd induces the self-protection mechanism of the body. However, significant decrease in activities of GSH-Px and CAT, accompanied by increase in MDA levels, demonstrated that the antioxidant system of the kidney was damaged in the relatively higher Cd dose groups.

In addition, we found that the activities of ATPase including Na^+^/K^+^-ATPase and Ca^2+^/Mg^2+^-ATPase decreased significantly in the relatively high Cd groups (30.55 and/or 60.67 mg/kg Cd). Similarly, Yuan et al. [50] reported that administration of Cd induced inhibition of Na^+^/K^+^- and Ca^2+^/Mg^2+^-ATPases activities of cerebral cortical neurons. Ca^2+^/Mg^2+^-ATPase, as a membrane-bound enzyme mainly found in plasma and mitochondrial and microsomal membranes, plays a crucial role in the maintenance of Ca homoeostasis in cells. Decrease of this enzyme may lead to increased intracellular Ca or reduced extracellular Ca influx, and ultimately to cell death [51]. Thévenod and Friedmann indicated that Cd-mediated oxidative stress in kidney proximal tubule cells induced degradation of Na^+^/K^+^-ATPase, which might contribute to the Fanconi syndrome-like Na^+^-dependent transport defects [42]. Therefore, Cd may compromise Ca homeostasis by interfering with ATPase activities in kidneys of laying hens.

## 5. Conclusions

The present study revealed that dietary Cd can induce renal dysfunction and oxidative stress, disrupt the secretion of estrogen and calciotropic hormones including PTH, CT and 1,25-(OH)2-D3, and damage the metabolism of the tibia of laying hens. Kidney dysfunction and especially tubular damage may be one of the main causes of bone damage. In birds, calcium derived from medullary bone is necessary during the final stages of shell formation. However, dietary Cd at a concentration of 60.67 mg/kg can deteriorate the balance of the medullary bone metabolism by increasing the resorption of trabecular bone, and ultimately affect the health of laying hens.

## Figures and Tables

**Table 1:** Ingredient compositions and nutrient levels of basal diet for hens.

Basal Ingredients	Value	Nutrient Level ^2^	Value
Corn, %	65	Metabolizable energy, MJ/kg	2.65
Soybean meal (42.0% crude protein), %	21	Crude protein, %	15.73
Feather meal, %	1	Ether extract, %	6.32
Fish meal, %	1	Lysine, %	0.78
Calcium carbonate, %	7	Methionine, %	0.34
Premix ^1^, %	5	Cysteine, %	0.32
		Total phosphorus, %	0.61
Total, %	100	Calcium, %	3.45

^1^ The premix provided the following per kilogram of diet: vitamin A, 7000 IU; vitamin D_3_, 2500 IU; vitamin E, 49.5 mg; vitamin K_3_, 1 mg; vitamin B_1_, 1.5 mg; vitamin B_2_, 4 mg; vitamin B_6_, 2 mg; vitamin B_12_, 0.02 mg; niacin, 30 mg; folic acid, 0.55 mg; pantothenic acid, 10 mg; biotin, 0.16 mg; chloride choline, 500 mg; sodium chloride, 2500 mg; Cu, 20 mg; Fe, 70 mg; Mn, 100 mg; Zn, 70 mg; I, 0.4 mg; Se, 0.5 mg. ^2^ Estimated from Chinese feed database provided with tables of feed composition and nutritive values in China (2015 26th edition).

**Table 2:** Effects of Cd on serum biochemical of renal function in laying hens.

Item	Dietary Cd Dosage, mg/kg					*p*-Value	SEM ^3^
	0.47	7.58	15.56	30.55	60.67		
Creatine, μmol/L	10.91 ^b^	16.81 ^ab^	14.85 ^ab^	13.33 ^ab^	21.47 ^a^	0.025	2.670
UA^1^, mg/L	22.64 ^c^	25.66 ^c^	27.07 ^bc^	32.37 ^ab^	35.22 ^a^	<0.001	1.794
BUN^2^, mmol/L	3.56 ^b^	3.52 ^b^	3.99 ^b^	4.67 ^ab^	5.82 ^a^	0.013	0.573

Values are presented as the mean and SEM (*n* = 8). ^a–c^ Means within a row with different superscripts are significantly different (*p* < 0.05). ^1^ UA, Uric acid; ^2^ BUN, blood urea nitrogen; ^3^ SEM, standard error of mean.
